# Frequency Dependent Non- Thermal Effects of Oscillating Electric Fields in the Microwave Region on the Properties of a Solvated Lysozyme System: A Molecular Dynamics Study

**DOI:** 10.1371/journal.pone.0169505

**Published:** 2017-01-27

**Authors:** Stelios Floros, Maria Liakopoulou-Kyriakides, Kostas Karatasos, Georgios E. Papadopoulos

**Affiliations:** 1 Faculty of Chemical Engineering, Department of Chemistry, Aristotle University of Thessaloniki, Thessaloniki, Greece; 2 Faculty of Health Sciences, Department of Biochemistry and Biotechnology, University of Thessaly, Mezourlo, Larisa, Greece; Brandeis University, UNITED STATES

## Abstract

The use of microwaves in every day’s applications raises issues regarding the non thermal biological effects of microwaves. In this work we employ molecular dynamics simulations to advance further the dielectric studies of protein solutions in the case of lysozyme, taking into consideration possible frequency dependent changes in the structural and dynamic properties of the system upon application of electric field in the microwave region. The obtained dielectric spectra are identical with those derived in our previous work using the Fröhlich-Kirkwood approach in the framework of the linear response theory. Noticeable structural changes in the protein have been observed only at frequencies near its absorption maximum. Concerning C_α_ position fluctuations, different frequencies affected different regions of the protein sequence. Furthermore, the influence of the field on the kinetics of protein-water as well as on the water-water hydrogen bonds in the first hydration shell has been studied; an extension of the Luzar-Chandler kinetic model was deemed necessary for a better fit of the applied field results and for the estimation of more accurate hydrogen bond lifetime values.

## Introduction

The dielectric properties of protein solutions have been the object of several studies over the years [1–10]. Although an increasing amount of knowledge has been accumulated regarding their dielectric response in a broad frequency range, the wide application of microwave technology in the telecommunication branch and in medical diagnosis and therapy [11–13] has made the study of the biological **non** thermal effects, specifically in the microwave band, of particular interest. The dielectric spectra of a protein can be understood generally in terms of three major relaxation mechanisms, the so called β-, γ- and δ- processes. The β-process (near 10^7^ Hz of the absorption curve) is attributed to the overall tumbling of the protein. The γ- process can be rationalized by the reorientation of bulk water and is centered near 10^10^ Hz. Finally the δ-relaxation process (near 10^8^−10^9^ Hz) is thought to arise either from the motions of protein-bound water molecules or from motions of charged side chains of the proteins [14]. Although the δ-process has only a small contribution to the absorption curve, it has drawn much attention [6, 7–10] because it is expected to provide new insight in protein-water interactions. In spite of the large number of dielectric experimental studies the molecular interpretation of the macroscopic results still puzzles the community, due to the fact that interesting mechanisms overlap in this region of the total absorption spectrum.

The atomic-level detail afforded by molecular dynamic simulations (MDS) [14–24] can help to resolve ambiguities related to the origins of these mechanisms. Usually the Fröhlich-Kirkwood (F-K) approach [25–30] and the linear response theory (LRT) [22, 24, 28, 31–36] are applied on MDS trajectories in order to calculate the frequency-dependent dielectric constants, by means of separating the individual contributions of a solution's components to the overall dielectric constant [14, 22, 24, 36]. In a limited number of non-equilibrium MDS studies on lysozyme, large structural changes have been reported using very intense electric fields and frequencies > 10^9^ Hz, higher than the protein absorption band (β-process). Specifically, a rapid loss of the triple-stranded β-sheet and 3_10_ helix have been observed at 100 GHz and 500 mV/Å [37] as well as perturbations of the hydrogen bonding network in the beta domain of the protein at 2.45 GHz and 50 up to 150 mV/Å [38]. However, very high field strengths may trigger a non linear response and saturation effects; a breakdown of the linear response theory can in turn be the reason for a disagreement between experimental and theoretically predicted dielectric spectra.

In the present work MDS have been used on a lysozyme solution with application of an external oscillating electric field at different microwave frequencies, low enough to preserve linearity between induced dipole moment **M** and electric field **E**. The resulted trajectories have been then used to calculate the corresponding values of the dielectric function and to compare them with those predicted using the F-K approach [14] and with those derived from experiments [1, 3–10]. Moreover, possible structural changes at frequencies and field intensities two orders of magnitude lower than those reported in the literature [37, 38] have been considered, as well as field effects on the dynamics of protein-water hydrogen bonds.

## Theoretical Background

### Dielectric function

Here we assume that the applied oscillating electric field is parallel to the z-axis and is homogeneous in the entire space occupied by the simulation cell. Commonly, the oscillating field is given by:

$${\text{E}}_{\text{z}}={\text{E}}_{0}{\text{e}}^{\text{-i}\omega \text{t}}$$ (1)

(Symbols have their usual meaning)

Now let us consider a system of m different components, each of them (k = 1,…, m) behaving as a dielectric material. Their induced dipole moments at frequency ω can be shown to follow the expression [25]:

$${\text{M}}_{\text{k}}\left(\omega ,\text{t}\right)={\text{A}}_{\text{k}}\left(\omega \right)\cdot \text{cos}\left(\omega \text{t}\right)+{\text{B}}_{\text{k}}\left(\omega \right)\cdot \text{sin}\left(\omega \text{t}\right)$$ (2)

The frequency dependent constants A_k_ and B_k_ are connected directly with the real and the imaginary part of the dielectric constant [25], via equations:

$$\text{Re}\left[\varepsilon_{\text{k}}\left(\omega \right)\right]-1=\frac{{\text{A}}_{\text{k}}\left(\omega \right)}{\varepsilon_{0}\text{V}\cdot {\text{E}}_{0}}$$ (3a)

$$\text{Im}\left[\varepsilon_{\text{k}}\left(\omega \right)\right]=\frac{{\text{B}}_{\text{k}}\left(\omega \right)}{\varepsilon_{0}\text{V}\cdot {\text{E}}_{0}}$$ (3b)

Here V is the simulation cell volume. The total dielectric constant of the system is simply given by the summation of all the above m dielectric terms, that is:

$$\varepsilon \left(\omega \right)=\text{Re}\left[\varepsilon \left(\omega \right)\right]-\text{iIm}\left[\varepsilon \left(\omega \right)\right]=\sum_{\text{k}=1}^{\text{m}}\text{Re}\left[\varepsilon_{\text{k}}\left(\omega \right)\right]-\text{i}\sum_{\text{k}=1}^{\text{m}}\text{Im}\left[\varepsilon_{\text{k}}\left(\omega \right)\right]$$ (4)

It is important to mention that in this work we do not include the dielectric behavior of ions. This is actually a common practice in dielectric relaxation spectroscopy experiments when a better resolution of the β, γ and δ-processes is required. This is usually realized by removing the DC conductivity of ions [6], i.e. by subtracting the term iσ_0_/E_0_ω from the dielectric spectra. Moreover, as we showed in a previous work [14], due to the small number of ions, their contribution to the dielectric constant is only minor and can be safely disregarded.

### Hydrogen bond dynamics

The dynamics of hydrogen bond (HB) formation between water and the protein residues as well among water molecules themselves, can be well characterized by two time correlation functions [39], the continuous hydrogen bond time correlation function S(t) and the intermittent hydrogen bond time correlation function C(t). These correlations functions are defined by the following equations:

$$\text{S}(\text{t})=\frac{\langle \text{h}(0)\cdot \text{H}(\text{t})\rangle }{\langle \text{h}(0)\rangle }$$ (5a)

$$\text{C}(\text{t})=\frac{\langle \text{h}(0)\cdot \text{h}(\text{t})\rangle }{\langle \text{h}(0)\rangle }$$ (5b)

Where h(t) and H(t) are two hydrogen bond population variables. Specifically h(t) is unity when a particular pair of sites is hydrogen bonded at time t and zero otherwise. On the other hand H(t) is unit when a specific pair remains continuously bonded from time 0 to time t. Thus S(t) describes the probability that a hydrogen bond has formed and remains stable at all times up to t (a strict definition of lifetime), while C(t) describes the probability that a tagged pair is hydrogen bonded at time t, given it was bonded at time zero, i.e. C(t) allows breaking and reformation of hydrogen bonds of a tagged pair at intermediate times.

Appropriate multi exponential fits on the above dynamic functions lead to the calculation of the so called intermittent τ_C_ and continuous τ_S_ average relaxation times of the protein-water or inter-water system. These time averages provide a good description of rotational and translational relaxation motions as well as of kinetic bond mechanisms in different parts of the system (especially in active sites and hydration shells).

Following the concepts and notation of reference [39] the kinetics of breaking and formation of protein-water bonds can be described as:

$$\text{B}\;\overset{{\text{k}}_{1}}{\underset{{\text{k}}_{2}}{↔}}\;\text{QF}$$ (6)

Where B and QF, are the bound and quasi-free state of a specific pair of sites that participate to form a hydrogen bond. Since QF is defined by a cutoff distance, in our implementation water molecules arrive to this state either by rotating around the protein site or around their center of mass. A simple rate equation for the “reactive flux” has the following form [40, 41, 42]:

$$\text{k}(\text{t})=-\frac{{\text{dC}}_{\text{PW}}(\text{t})}{\text{dt}}={\text{k}}_{1}{\text{C}}_{\text{PW}}(\text{t})-{\text{k}}_{2}{\text{N}}_{\text{PW}}(\text{t})$$ (7)

Where k_1_ and k_2_ are the forward (breaking) and backward (re-formation) rate constants for the bound and quasi-free state respectively. C_PW_(t) is the intermittent hydrogen bond time correlation function C(t) in case of protein-water HBs. N_pw_(t) is a time correlation function that takes into consideration the diffusion of water molecules and can be calculated by the formula:

NPW(t)=〈h(0)⋅[1−h(t)]⋅H'(t)〉〈h(0)〉 (8)

Where H'(t) is unity when a pair of sites is closer than a cutoff distance R_PW_ (usually taken equal to 3.2 Å) for protein-water interactions at time t and zero otherwise. I.e., the N_pw_(t) correlation accounts for water populations which are not any more hydrogen bonded to protein but remain close enough to reform a broken bond. The above model is first introduced by Luzar and Chandler (L&C) [40–42].

#### Extension of luzar and chandler model of hydrogen bonds kinetics

In this section we modify Eq (6) in order to include an additional relaxation process. This modification is necessary to describe better our simulation results related to the influence of the electric field on the hydrogen bonds kinetics. The calculation of the dynamic functions starts considering the instant configuration of the hydrogen bond network between protein and its neighboring water molecules, which defines the t = 0. For the above system one can write the following double reversible reaction:

$$\text{QF}\prime \;\overset{{\text{k}}_{1}^{\prime }}{\underset{{\text{k}}_{2}^{\prime }}{↔}}\;\text{B}\;\overset{{\text{k}}_{1}}{\underset{{\text{k}}_{2}}{↔}}\;\text{QF}$$ (9)

**Fig 1:**
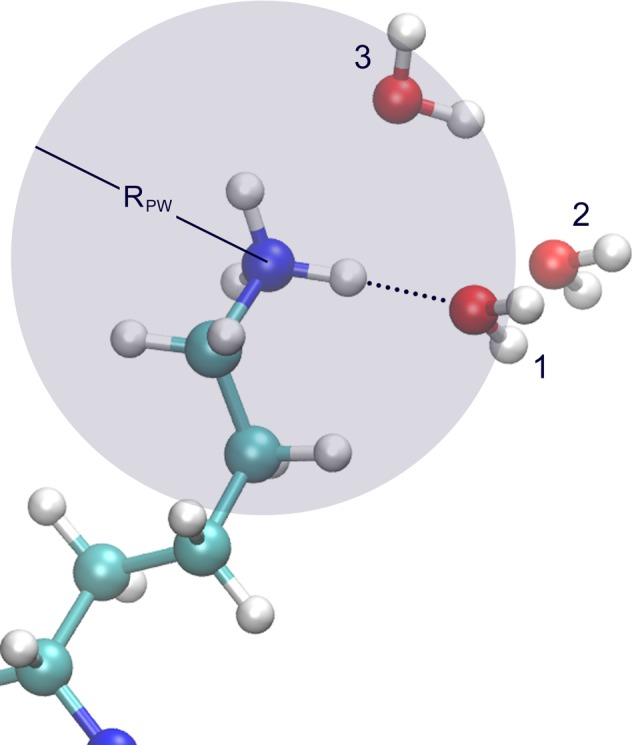
An Arginine side chain in hydrogen bonding (B) configuration. At t = 0 a water molecule may occupy position 1, lying in the range of R_PW_ (= 3.2 Å) around a polar site. On a next simulation frame the water molecule may occupy the non bonding position 3 (QF) or may be found slightly beyond R_PW_ designated as QF' water.

Following the fate of each previously bound water molecule, the left part of reaction (9) describes the direct transition from B to a second quasi free state QF' and vice versa (Fig 1). That is, we consider the possibility of some originally bound water molecules to be found slightly beyond the cut-off distance R_PW_ from a specific protein site after the initial breaking. On the other hand QF' represents a second "activated state" along a reaction coordinate defined by the distance (very close to R_PW_) from the protein site allowing some water molecules to escape, while others to directly reform the HB. In our calculations we consider a limit for this distance determined by the largest distance R_max_ observed after the first bond breaking, which is actually related to the frame recording rate. All previously bound water molecules found in a spherical shell between R_PW_ and R_max_ belong to the small QF' population not captured by the L&C model. The right side of reaction (9) refers to the transition between the bound B and quasi-free state and vice versa. According to model (9) a “reactive flux equation” can be written for C_PW_ similar to Eq (7):

$$\text{k}(\text{t})=-\frac{{\text{dC}}_{\text{PW}}(\text{t})}{\text{dt}}={\text{k}}_{1}{\text{C}}_{\text{PW}}(\text{t})-{\text{k}}_{2}{\text{N}}_{\text{PW}}(\text{t})-{\text{k}}_{2}^{'}{\text{F}}_{\text{PW}}(\text{t})$$ (10)

where k_1_ accounts for the total (k_1_+k'_1_) forward (breaking) of HBs of model (9). F_PW_(t) is a function introduced to describe the time correlation of QF' water molecules. The F(t) correlation is given by:

$$F_{PW}(t)=\frac{\langle h(t_{0})\cdot [1-h(t_{0}+t)]\cdot [1-H^{\prime }(t_{0}+t)]\cdot Q\left(t_{0},R_{PW}<r\leq R_{\max}\left(t_{0}\right)\right)\rangle }{\langle h\rangle }$$ (11)

Where h(t) and H^’^(t) are the previously defined hydrogen bond population variables. Function Q takes at time t the value 1 if there is at least one water molecule (after an initial HB breaking) at distance R_PW_ < r ≤ R_max_(t_0_) from a protein’s site able to reform directly the previous broken hydrogen bond. Here t_0_ defines the starting simulation frame for the correlation analysis. Since we count hydrogen bonds using labeled water molecules from concrete populations, their numbers will become gradually less and less resulting to a relaxation of the corresponding HBs correlation functions C(t), N(t) and F(t). According to our findings, the first hydration shell is less dense under field conditions than without. Therefore, water molecules in the QF' state have a smaller probability to defuse away than under zero field conditions allowing for a higher HBs reformation probability. It is this behavior which we incorporated in the reactive flux equation. As it will be shown in section “Results and Discussion” the incorporation of the F dynamics into the basic kinetic formula of L&C leads to better least square fits and consequently to more accurate calculations of average lifetimes especially in the case of electric field induced effects.

In this work k(t) is calculated either as -ΔC/Δt from the simulation data giving k_sim_, or from a regression fit of two independent variables (C, N) adjusting the rate constants k_1_, k_2_ to satisfy Eq (7), resulting to k_LC_. When determined from a three variables (C, N, F) fit to satisfy Eq (10) k(t) is named k_F_. From the rate constant k_1_ determined by the fits to Eqs (7) or (10) we can then calculate relaxation times ζ = 1/k_1_ for protein-water (PW) and inter-water (WS1) hydrogen bond breaking.

## Simulation Details

A series of MDS has been performed aiming to a) the determination of the dielectric spectrum of a lysozyme solution with a direct method (application of an oscillating electric field at different frequencies of the spectrum) and b) to the study of possible non thermal effects of microwave radiation on lysozyme’s structural and dynamical features.

As reported in our previous work [14], the starting complete structure of lysozyme (PDB entry: 2LZT) has been solvated in an orthogonal box (48.10×52.40×66.65 Å^3^) with 4672 TIP3P water molecules giving a protein concentration of 9.88 mM (w/w = 14.5%, mass of H_2_O over dry protein = 5.88). Disulphide bonds have been added at Cys6-Cys27, Cys30-Cys115, Cys64-Cys80 and Cys76-Cys94. The positive charge of lysozyme (+8) has been neutralized by the addition of ions (18 Na^+^ and 26 Cl^-^). The molecular dynamics program NAMD [43] and the CHARMM force field [44] have been used to energy minimize and pre-equilibrate the system for 2.5 ns followed by a production run of 30 ns with Langevin thermostat at 300 K in the NVT ensemble. The SHAKE algorithm [45] has been applied to all bonds and interactions were calculated with a cut-off radius of 16 Å. Electrostatic interactions were calculated with the Particle Mesh Ewald technique [46] while assuming tin-foil boundary conditions. The time step of the simulations was 2 fs and configurations of the system were stored every 0.5 ps.

Starting from the fully equilibrated state the above system was exposed during simulation to a uniform internal cosinusoidal electric field (E_0_ = 4.2 mV/Å) at 21 frequencies in the range of 10^7^ to 10^11^ Hz using the eField parameters in the NAMD configuration file [47]. For the sake of comparison, the applied field is in the same order of magnitude as the field across a biological membrane (~1 mV/Å), while it is five orders of magnitude higher than that emitted by a cell phone (~10^-5^mV/Å). However such high intensities are necessary for small effects to exceed the thermal noise within the limited time scales generally amenable to molecular simulations. Field effects, like those presented later in this study, may arise as result of much lower intensities after a long time (minutes or hours) exposition. Unfortunately, such time domains are currently inaccessible to MDS. The simulations were performed in the NVT ensemble at 300 K. The computational time of each simulation was frequency dependent, i.e. for the lowest frequency of 10^7^ Hz a total simulation time of almost 100 ns was necessary in order to cover a full cycle of the field (data saved every 2 ps). With increasing frequency the simulation time was decreased reaching a value of 0.01 ns at 10^11^ Hz (data saved every 20 fs). For each frequency above 10^9^ Hz twenty independent simulations were carried out in order to improve the statistics for our calculations.

Prior to applying a frequency to the protein solution the highest field strength value has been determined for which the system responds linearly. This is necessary in order to avoid break down of the linear response theory. A series of simulations have been performed with a static electric field applied on a system consisting of water molecules. The field amplitude has been increased starting from a value of 0.42 mV/Å and the upper acceptable limit for which the total dipole moment of water responds linearly has been determined to be 4.2 mV/Å. This value has been adopted for further frequency dependent simulations.

The simulation data have been analyzed using home made Tcl scripts running on the VMD program [48]. Further, the Gnuplot program has been used for fitting of the respective curves.

## Results and Discussion

### Dielectric calculations based on application of oscillating electric fields

**Fig 2:**
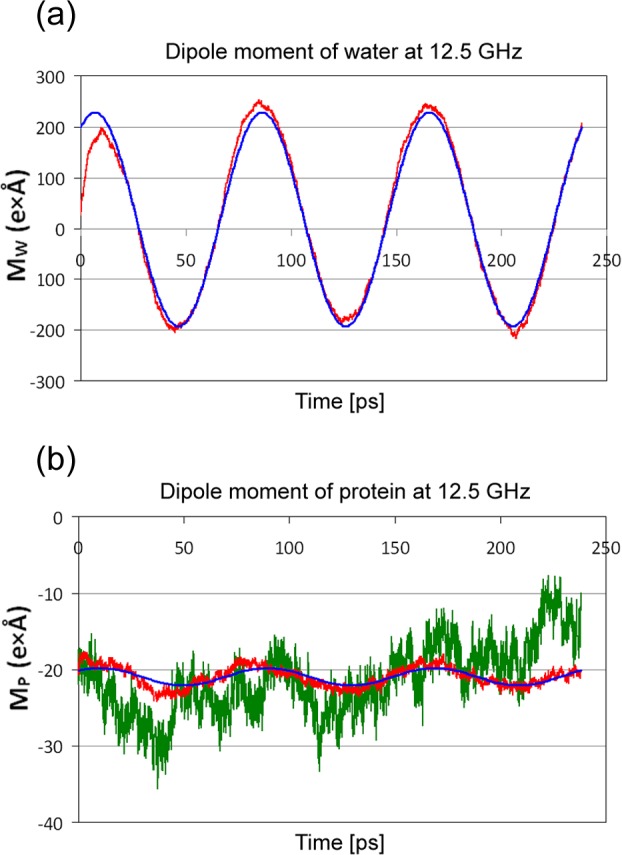
Dipole moment response of water and protein to a 12.5 GHz field. (a): The z-component of the total dipole moment M_W_ of water (red line) during three periods of applied electric field at 12.5 GHz and appropriate fit (blue line) of the water dipole moment to Eq (2). (b): The z-component of the total dipole moment of protein (green line) during three periods of applied electric field at 12.5 GHz as derived from only one simulation. The protein dipole moment (red line) determined as an average over 20 independent simulations, which drastically reduced thermal noise. Appropriate fit (blue line) of the protein dipole moment to Eq (2).

**Fig 3:**
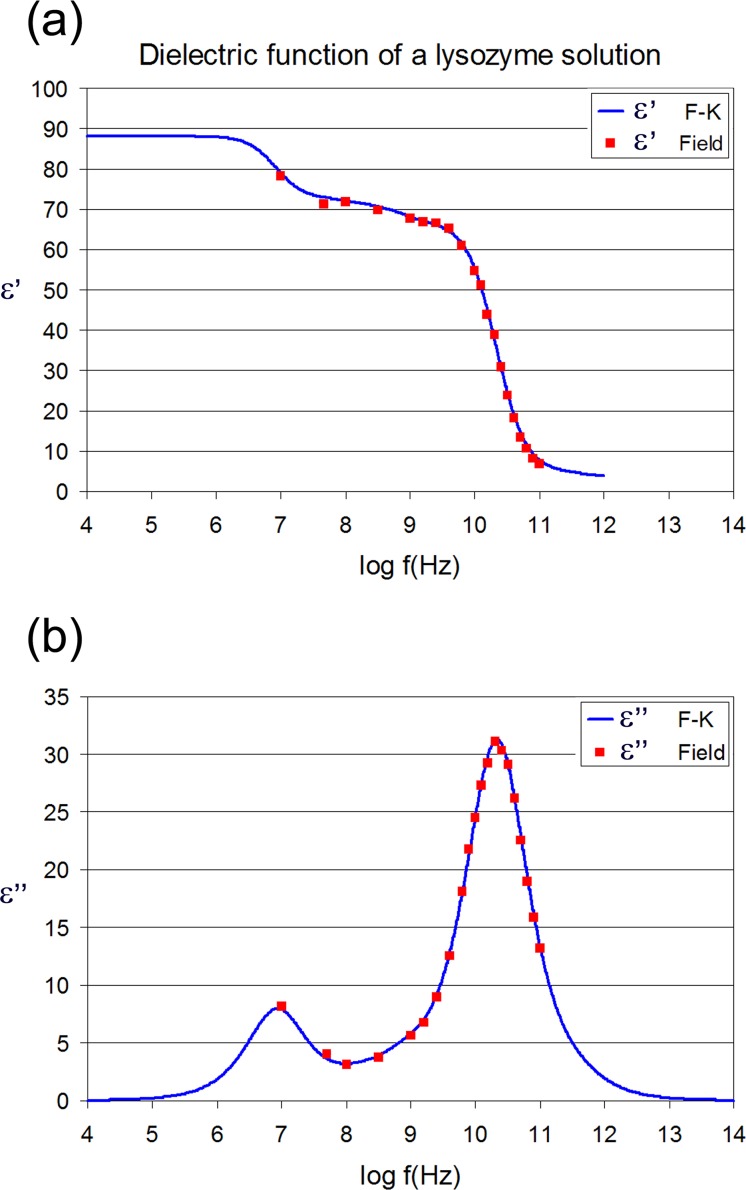
Calculated dielectric function of a lysozyme solution. System composition and simulation conditions are as in [14]. The real (a) and imaginary (b) part of the dielectric function of the entire system as calculated by the F-K approach (blue line) as well as by applying an oscillating field (red squares).

**Fig 4:**
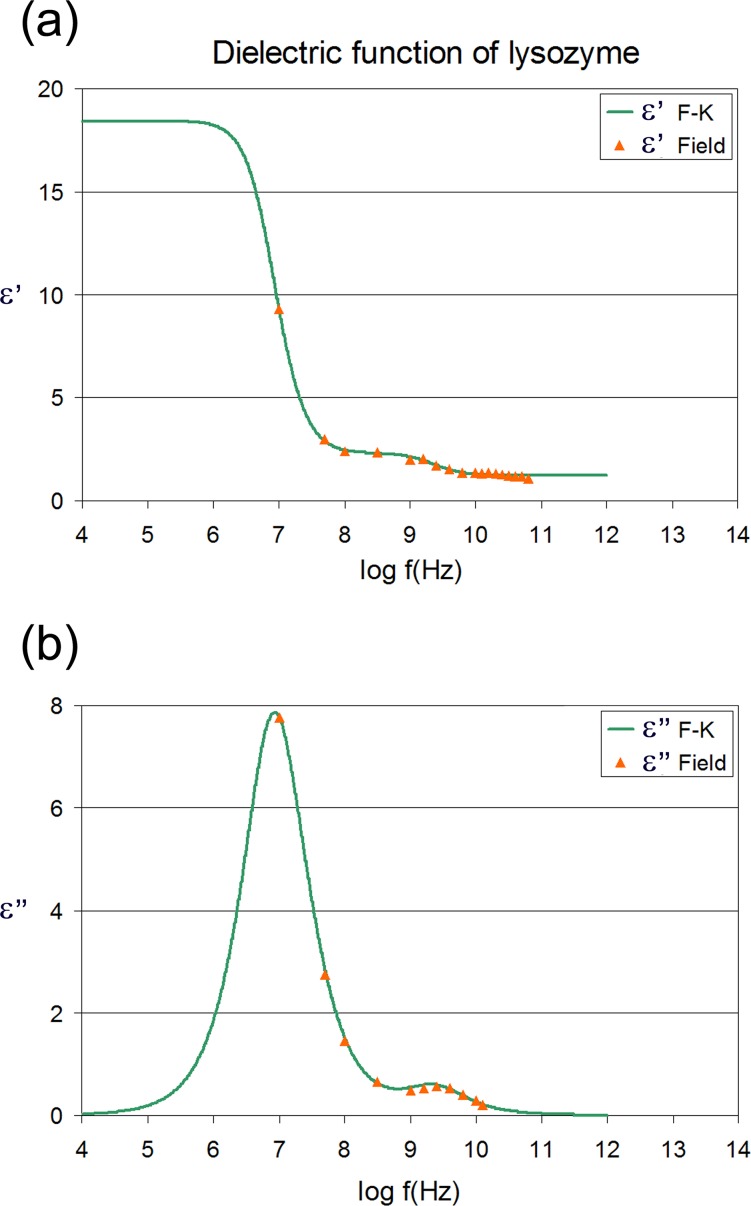
Calculated dielectric function of lysozyme. System composition and simulation conditions as in [14]. The real (a) and imaginary (b) part of the dielectric function of the protein as calculated by the F-K approach (green line) as well as by applying an oscillating field (orange triangles).

The dipole moment of the system components has been calculated for each one frame of the simulation trajectories derived for different applied frequencies of the dielectric spectrum. Though a field amplitude of 4.2 mV/Å is low enough to guaranty linearity, the price to pay is a low signal to noise ratio in the case of a protein. It is apparent from Fig 2A that the z-component of the dipole moment response of the total water (red line) at 12.5 GHz is periodic with time, giving a very good signal compared to noise. A fit to Eq 2 (blue line), provided the values for A_k_(ω) and B_k_(ω) which were then introduced in Eqs (3a) and (3b) to calculate the real and imaginary part of the dielectric function at this frequency. The fact that 12.5 GHz is at the absorption maximum of water, but far enough from that of protein, leads to a different behavior of the protein (Fig 2B). Apparently, in the case of only one simulation at this frequency (green line) the statistic is too poor to reveal a clear signal, since thermal motion of the surface residues overcomes the response to an electric field of the given strength. For this reason 20 independent simulations have been averaged (red line) reducing drastically the noise. This allowed an acceptable fit (blue line) to the analytic function of Eq (2). It should be noted that the number of simulations needed to eliminate noise depended on the frequency and decreased gradually with decreasing frequency. Only one simulation is performed at 10^7^ Hz, with a total simulation time of 100 ns for one full field cycle. This procedure spanned the dielectric function of the system and its components over a frequency range of 10^7^–10^11^ Hz (Figs 3 and 4).

The calculated dielectric functions of a lysozyme solution and of protein alone are illustrated in Figs 3 and 4 respectively. Blue lines represent the dielectric function as calculated by the F-K approach and LRT according to a detailed decomposition method [14], while red squares represent values of the frequency dependent real and imaginary parts of the dielectric constant, as calculated by applying an oscillating electric field. In the case of protein alone the chromatic code is green lines and orange triangles (see Fig 4). Both, the locations and the heights of the peaks practically coincide. Moreover, concerning the δ-relaxation process (10^8^−10^9^ Hz) the agreement is very good too, supporting the bi-modal nature of its dispersion curve [6, 14]. Our calculations of the dielectric function by applying oscillating fields shows that the spherical protein approximation in the Fröhlich-Kirkwood approach works very well in the case of an ellipsoidal protein like lysozyme and keeping the field intensity lower than the saturation limit. Differences in the calculated dielectric spectra were expected in studies of proteins of shapes deviating rather largely from the spherical symmetry. However, in order to confirm the later statement, more studies with different proteins have to be performed. This is a key result, because it demonstrates the ability to calculate the dielectric properties of proteins from atomistic simulations without the assumption of an almost spherical symmetry assumed by the F-K approach. Moreover, the versatility of this non-equilibrium approach (at least for field strengths as high as the one used here) allows the study of the field effects on various protein structural and dynamic properties, as will be discussed in the next section.

### Field effects on protein structure

In this section the effect of the applied field on protein's structure will be examined in detail. Protein structural changes will be considered, as reflected in the time course of protein’s RMSD (C_α_ root mean square deviation relative to lysozyme's crystal structure), in the RMSF (root mean square fluctuations) of individual residues and in the % of protein’s secondary structure.

**Fig 5:**
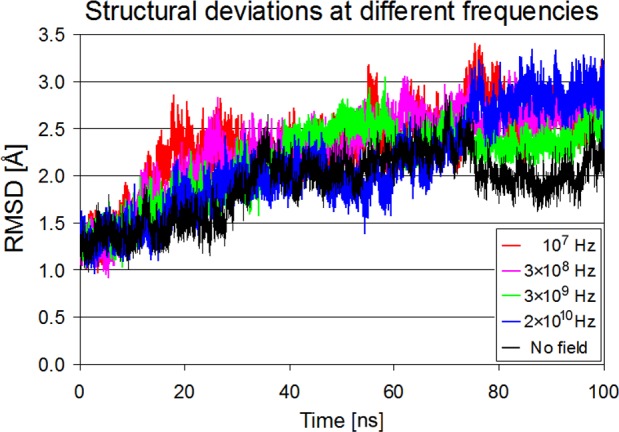
Lysozyme structural deviations vs. simulation time at different field frequencies. RMSD values of protein’s C_α_-atoms with a field amplitude of 4.2 mV/Å at 10^7^Hz (red), 3×10^8^ Hz (purple), 3×10^9^ Hz (green), 2×10^10^ Hz (blue) and with zero field conditions (black) at 300 K. All RMSDs are calculated relative to the crystal structure.

Fig 5 displays the time evolution of protein’s backbone deviation from the crystal structure at four frequencies (f_1_ = 10^7^ Hz, f_2_ = 3×10^8^ Hz, f_3_ = 3×10^9^ Hz and f_4_ = 2×10^10^ Hz) and at zero field. Frequency f_1_ of the main protein absorption as well as f_2_ next to it, exhibit protein structure deviations from the crystal structure relatively early (after 20 ns), while f_3_ after about 40 ns. On the other hand, frequency f_4_ of the bulk water absorption, well beyond the main absorption maximum of protein, shows a delayed (after 70 ns) but significant structural deviation larger than that appeared at f_3_ and similar to that characterizing f_1_. According to references [37] and [38] the application of more intense oscillating fields (50 or 500 mV/Å) can lead to a dramatic increase in the RMSD values at frequencies away from the β-process zone.

**Fig 6:**
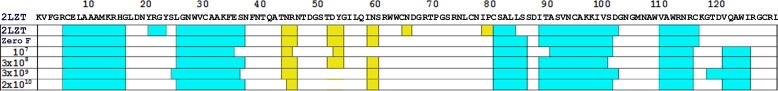
Secondary structure of lysozyme at different frequencies and zero field. Distribution of secondary structure elements over the lysozyme’s sequence at different frequencies (Hz), at the end of 100 ns trajectories and zero field conditions, as detected by VMD. Cyan: Helix, Yellow: β-strand. 2LZT stands for the PDB notation of the protein’s crystal structure.

**Fig 7:**
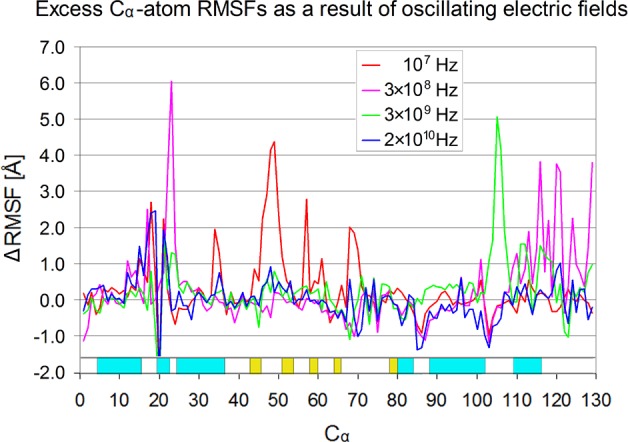
Excess average backbone fluctuations along the sequence of lysozyme. Excess C_a_-atom fluctuations of lysozyme at 10^7^Hz (red), 3×10^8^Hz (purple), 3×10^9^Hz (green) and 2×10^10^ Hz (blue) (E_0_ = 4.2 mV/Å, T = 300 K). ΔRMSFs (excess fluctuations) at different frequencies resulted after subtraction of the C_a_ RMSFs without field. Cyan and yellow bars above the C_α_ numbering designate helix and β-strands of lysozyme’s crystal structure.

In relation to secondary structure (Fig 6), VMD recognizes in the crystal structure six helical segments, helix-1: Arg5—His15, helix-2: Tyr20—Gly22, helix-3: Leu25—Ser36, helix-4: Cys80—Leu83, helix-5: Ile88—Asp101 and helix-6: Val109—Cys115 as well as five beta segments, strand-1: Thr43—Arg45, strand-2: Thr51—Tyr53, strand-3: Ile58—Asn59, strand-4: Cys64—Asn65 and strand-5: Ile88—Pro89. At the end of the simulation without field the same software recognizes helix-1, helix-3, helix-4, helix-5 and helix-6 and three beta conformations, strand-1, strand-2 and strand-3. A comparison of protein’s secondary structure before and after exposure to the field at different frequencies (Fig 6) reveals changes in the amino acid ranges both of helices and β-strands without affecting significantly the overall structural organization of lysozyme. Specifically, all simulations lack short helix-2 and β-strands -4 and -5. On the other hand, it seems that field conditions promote the formation of helix-7 which is absent in the crystal structure and without field. Moreover, strand-3 is missing at 10^7^ Hz, while strand-2 is missing at 3×10^9^ and 2×10^10^ Hz. Some elongation and shortening effects can also be observed. Frequency f_1_ destabilizes (Fig 7) the right end of helix-3 including Glu35 and the left end of strand-2 including Asp52, both being the key catalytic residues of lysozyme, as well both ends of helix-5 (Ile88—Asp101). Table 1 summarizes the alterations in secondary structure content—beside radius of gyration (R_g_) and RMSD. Since the numbers given in this table are averages over 5000 values the standard errors of the mean are very small (< 0.034). It is obvious that the field promoted the helical conformation in the C-terminus. The above changes in protein’s secondary structure had no effect on the radii of gyration and do not go hand in hand with effects on RMSD. Besides, the largest RMSD is observed at 2×10^10^ Hz, which is the main absorption frequency of bulk water. More substantial structural changes may be expected in proteins with less disulphide bonds.

**Table 1:** Alterations in percentage of secondary structure content, radius of gyration (R_g_) and RMSD of lysozyme due to application of an oscillating field at 10^7^ Hz, 3×10^8^ Hz, 3×10^9^ Hz and 2×10^10^ Hz compared to zero field conditions and the crystal structure. All parameters are calculated as averages over the last 1ns (5000 frames, every 0.2 ps) of the corresponding 100 ns simulations.

f [Hz]	% Helix (SD)	% Beta (SD)	R_g_ (SD) [Å]	*RMSD [Å]
2LZT	45.74	10.85	14.07	0.00
**Zero field**	39.70 (2.00)	7.66 (0.35)	14.82 (0.11)	2.06 (0.16)
**f**_**1**_ **= 10**^**7**^	38.49 (2.05)	5.71 (0.65)	14.56 (0.08)	2.63 (0.15)
**f**_**2**_ **= 3×10**^**8**^	38.87 (2.21)	7.86 (0.48)	14.69 (0.09)	2.72 (0.12)
**f**_**3**_ **= 3×10**^**9**^	43.01 (1.33)	7.67 (0.59)	14.11 (0.06)	2.43 (0.12)
**f**_**4**_ **= 2×10**^**10**^	41.54 (2.42)	7.34 (0.85)	14.72 (0.14)	2.88 (0.14)

*RMSDs are calculated relative to the crystal structure of lysozyme (PDB:2LZT).

SD: Standard deviation. The statistical significance has been calculated for differences between the average values of Table 1 and those under zero field conditions. All differences are found to be statistically significant (P-Values < 0.00001) at a significance level < α = 0.001, except for %Beta at 3×10^9^ Hz (P-Value = 0.30301).

Considering the above information, it is interesting to see which protein regions experience the most significant structural fluctuations under the influence of the oscillating field and if there is a frequency effect. This is shown in Fig 7, which depicts the excess C_α_-atom fluctuations ΔRMSF (after subtracting the zero field RMSFs) at frequencies f_1_, f_2_, f_3_ and f_4_. Interestingly, different frequencies affect different protein segments and the most striking fluctuations are located in loop regions, except one on the right end of helix-2 (Lys25—Ser36) triggered by f_1_ and which contains Glu35, an important residue for the catalytic function of lysozyme. This observation differentiates from findings corresponding to more intense fields [38], where RMSF curves of 10 mV/Å at 2.45 GHz are found to be almost identical relative to zero field conditions. On the other hand, in case of an even higher and static field (50mV/Å) two charged segments (Thr43—Ile55 and Asn65—Leu75) showed high perturbations and disruption of hydrogen bonding, while segment Leu75 to Leu129 was not affected. According to this picture, the binding affinity of lysozyme to its substrate could be affected by the field at frequency f_1_. An indication of such an effect can be distinguished in the enhanced mobility of Phe34 (ΔRMSF ≈ 2 Å) and Leu56 (ΔRMSF ≈ 2.7 Å) which belong to the substrate’s binding cleft.

The structural effects described above combined with effects of the field on the intra-protein and protein-water hydrogen bonds -next in the text- may be correlated to effects of long exposure to cell phone radiation on enzyme activity, as observed in the case of acetylcholinesterase [49].

### Hydrogen bond analysis

Next, the number and the dynamics of intra-protein (PP), protein-water (PW) and inter-water (WS1) hydrogen bonds in the first hydration shell (S1) will be discussed in detail. Here the realization of HBs has to satisfy the following geometric criteria: a cutoff distance of 3.2 Å for PW HBs and 3.5 Å for WS1 and PP HBs, while the angle formed by the donor, hydrogen, and acceptor is within 150–180 degrees.

#### Hydrogen bond dynamics

**Fig 8:**
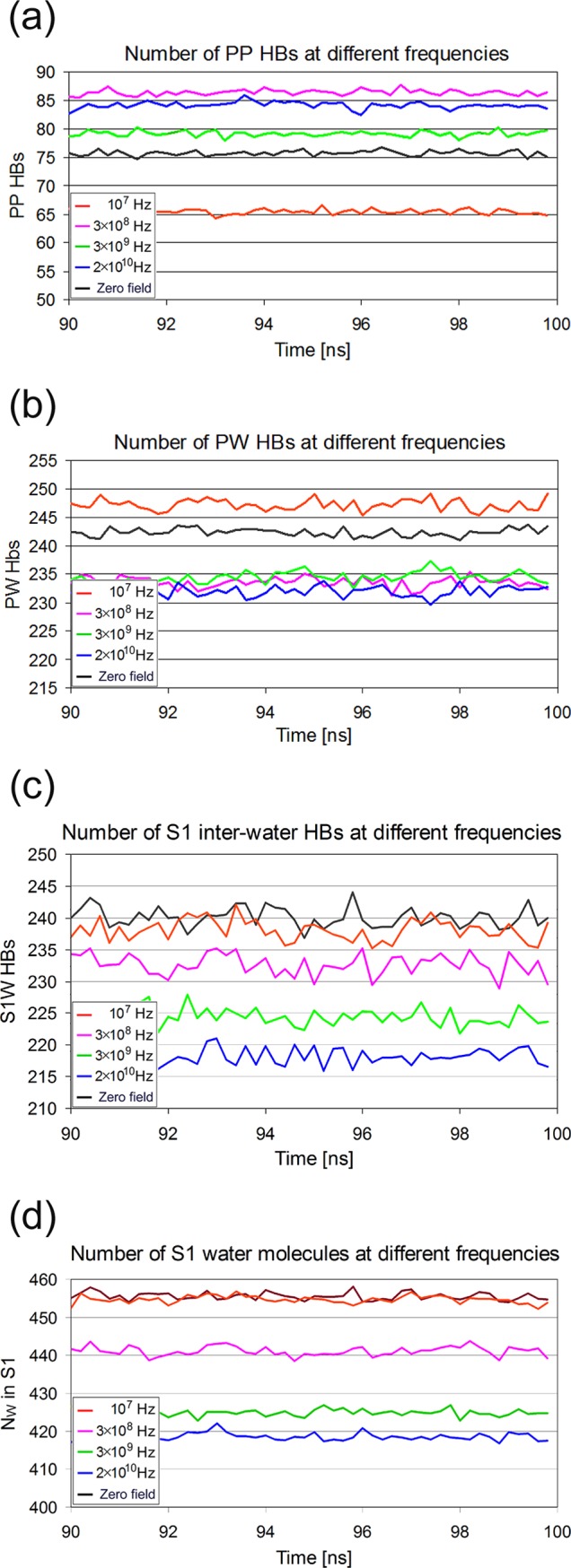
Field effects on the number of hydrogen bonds and S1 water molecules. Number of hydrogen bonds and S1 water molecules during the last 10 ns of 100 ns simulations in oscillating electric fields at f_1_ = 10^7^ Hz (β-process), f_2_ = 3×10^8^ Hz, f_3_ = 3×10^9^ Hz (δ_2_-process), f_4_ = 2×10^10^ Hz (γ-process) and without field. (a) Intra-protein HBs. Frequency f_1_ has a clear negative effect on the number of intra-protein HBs, while f_2_ and f_4_ a small positive one. (b) Protein-water HBs. The oscillating electric fields reduce the number of protein-water HBs. (c) Water-water HBs in S1. The field decreases the number of inter-water HBs. (d) Number of S1 water molecules. Frequencies f_2_, f_3_ and f_4_ decrease the number of water molecules.

**Fig 9:**
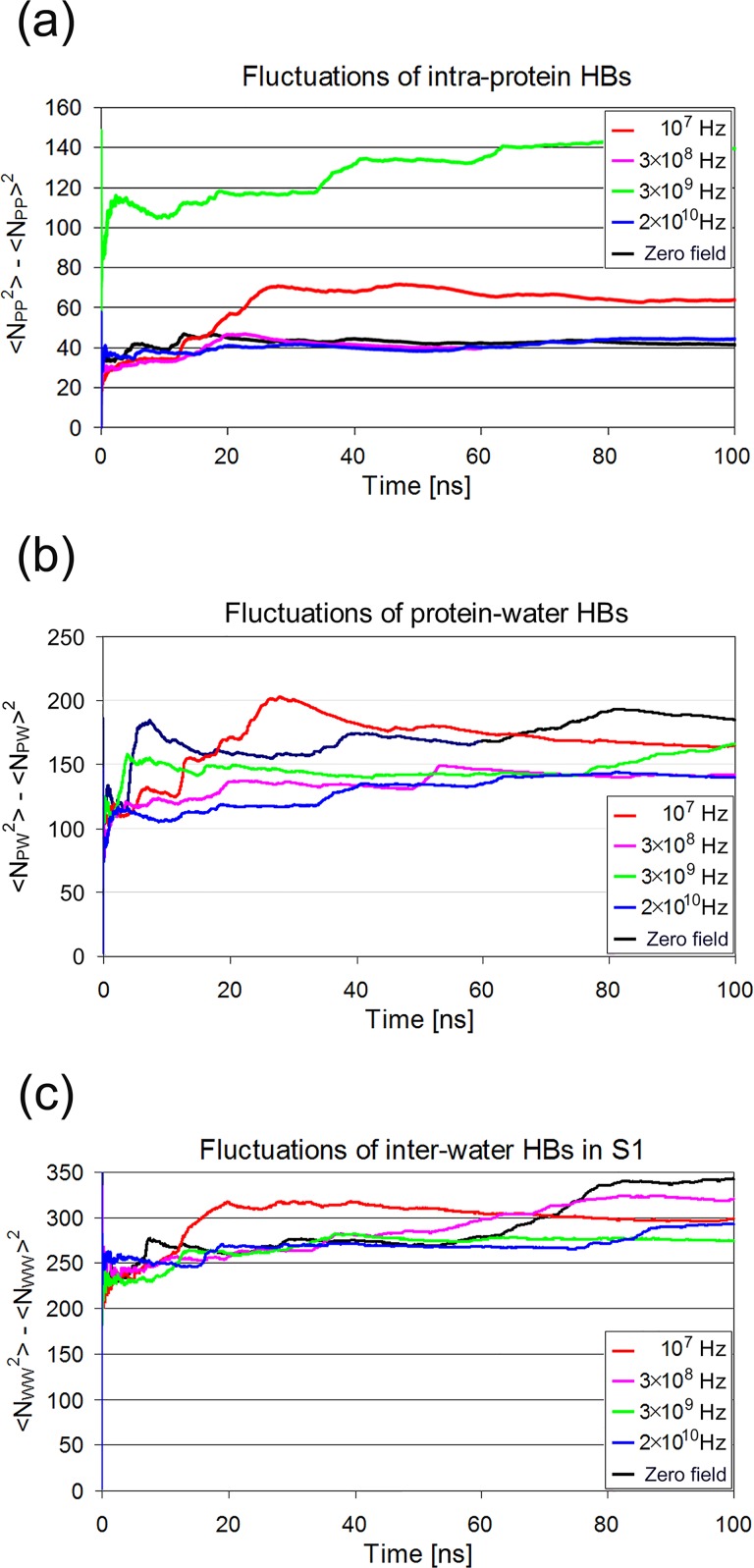
Fluctuations of the number of HBs with time at four frequencies and without field. (a) Intra-protein HBs. At f_4_ and zero field the fluctuations converge earlier and to lower values. (b) Protein-water HBs fluctuations at four frequencies show different convergence tendencies. (c) Fluctuations of HBs between water molecules in the S1 solvation shell do not show analogous convergence tendencies to (a) and (b).

**Fig 10:**
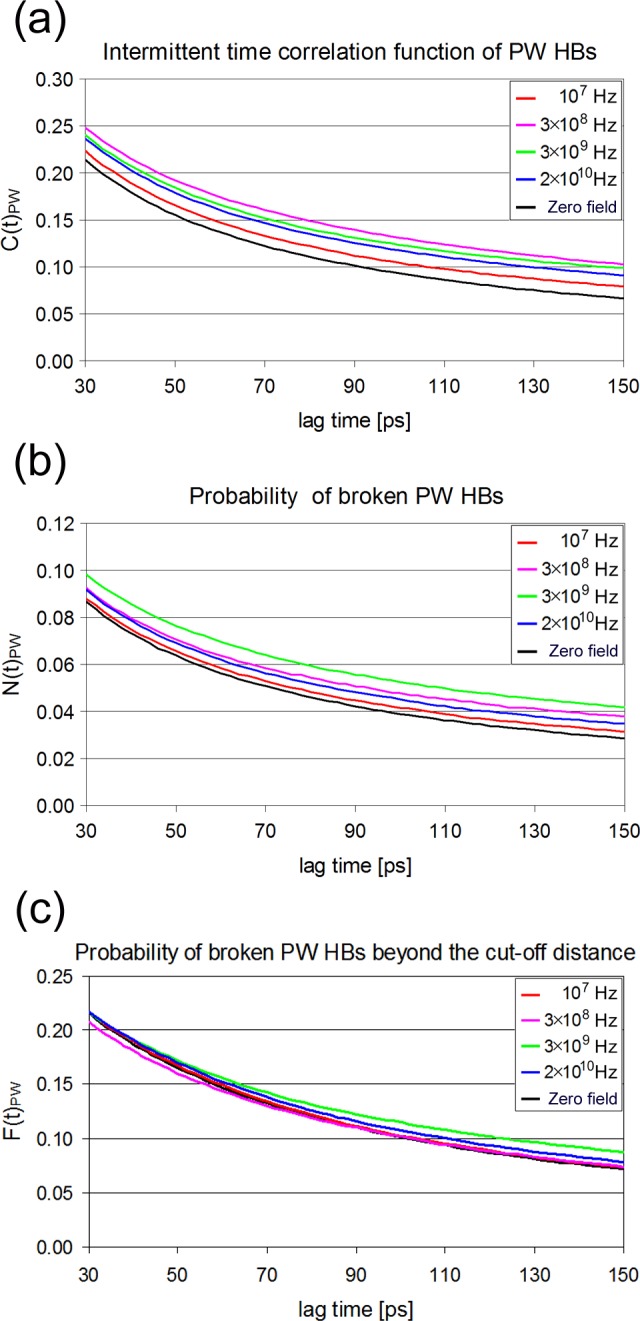
Correlation functions characterizing protein-water hydrogen bond breaking at different frequencies at 300 K. For better visualization of the differences at longer times data are plotted between 30 and 150 ps. (a) Intermittent hydrogen bond time correlation function. (b) Correlation probability functions of broken hydrogen bonds. (c) Correlation probability functions of broken hydrogen bonds between the protein and water molecules located slightly beyond the cut-off distance.

The number of hydrogen bonds in different subsystems of the lysozyme solution does not seem to be affected dramatically by the oscillating electric field at any frequency studied here (Fig 8). However, the small differences observed (Table 2) between the average number of hydrogen bonds over the last 10 ns of the 100 ns trajectories can be considered statistically significant, because of the relatively large number of the samples used (5000 values each). The most notable effect is observed in frequency f_1_ (10^7^ Hz), where it appears that the number of intra-protein hydrogen bonds decreased (14.3%) relative to zero field conditions. An opposite effect (increase) is shown for higher frequencies, with the largest (13.1%) observed at f_2_ (3×10^8^ Hz). Eventually, field effects on hydrogen bonding patterns become more prominent if one considers the number fluctuations of HBs (Fig 9) in conjunction with their dynamics (Fig 10, Tables 2–4). Fluctuations are calculated as time dependent variances of the number of HBs. A cursory glance of Fig 9A reveals a strong effect of frequencies f_1_ and f_3_ on the intra-protein HBs fluctuations, while fluctuations at f_2_ and f_4_ do not deviate from zero field conditions.

**Table 2:** Average number N_W_ of water molecules in S1. Average number of PP, PW and WS1 HBs. Life times ζ_F_^PW^ and ζ_F_^WS1^ of PW and WS1 hydrogen bonds.

f (Hz)	N_W_ in S1 (SD)	PP HBs (SD)	PW HBs (SD)	WS1 HBs (SD)	ζ_F_^PW^ [ps]	ζ_F_^WS1^ [ps]
**Zero field**	454.9 (12.8)	75.7 (5.9)	242.3 (12.1)	240.1 (16.7)	2.96	1.96
**f_1_ = 10^7^**	453.3 (13.7)	65.4 (5.5)	247.2 (12.3)	238.1 (16.3)	2.45	1.46
**f_2_ = 3×10^8^**	442.2 (13.6)	86.3 (5.6)	233.3 (11.4)	232.6 (16.8)	0.88	2.03
**f_3_ = 3×10^9^**	424.4 (12.5)	79.1 (5.6)	234.4 (11.0)	224.4 (16.1)	2.44	1.65
**f_4_ = 2×10^10^**	418.4 (12.4)	84.1 (5.8)	231.8 (10.9)	218.0 (16.4)	1.08	2.09

Averages and standard deviations (SD) are calculated over the last 10 ns of the 100 ns trajectories (5000 values, every 2 ps). HB life times are calculated as ζ = 1/k_1_. The statistical significance has been calculated for differences between the average values of Table 2 and those under zero field conditions. All differences are found to be statistically significant (P-Values < 0.0001) at a significance level < α = 0.001.

**Table 3:** Weighted average relaxation times τ^PW^ of PW HBs correlation functions C_PW_(t), N_PW_(t) and F_PW_(t) at four characteristic frequencies and at zero field as well as hydrogen bond breaking rate constants k_1_, k_2_ and k'_2_.

f [Hz]	τ_C_^PW^ [ps]	τ_N_^PW^ [ps]	τ_F_^PW^ [ps]	k_1_^PW^ [ps^-1^]	k_2_^PW^ [ps^-1^]	k'_2_^PW^ [ps^-1^]
**Zero field**	34.4	14.9	43.7	0.338	0.697	0.036
**f_1_ = 10^7^**	42.2	16.7	47.6	0.408	0.878	0.049
**f_2_ = 3×10^8^**	56.4	20.5	48.1	1.138	2.777	0.067
**f_3_ = 3×10^9^**	52.2	22.4	60.4	0.410	0.740	0.120
**f_4_ = 2×10^10^**	47.6	17.9	52.0	0.929	2.293	0.031

Rate constants k_1,_ k_2_ and k'_2_ are adjusted to satisfy rate Eq (10) which includes the F(t) correlation of the free water molecules.

**Table 4:** Relaxation times τ of S1 HBs correlation functions C_WS1_(t), N_WS1_(t) and F_WS1_(t) at four characteristic frequencies and at zero field as well as hydrogen bond breaking rate constants k_1_, k_2_ and k'_2_.

f (Hz)	τ_C_^WS1^ [ps]	τ_N_^WS1^ [ps]	τ_F_^WS1^ [ps]	k_1_^WS1^ [ps^-1^]	k_2_^WS1^ [ps^-1^]	k'_2_^WS1^ [ps^-1^]
**Zero field**	5.1	10.6	1.7	0.51	0.18	0.04
**f_1_ = 10^7^**	6.9	11.4	1.2	0.69	0.31	0.05
**f_2_ = 3×10^8^**	5.3	11.6	1.8	0.49	0.19	0.03
**f_3_ = 3×10^9^**	8.1	13.8	1.4	0.61	0.30	0.03
**f_4_ = 2×10^10^**	5.8	11.7	1.6	0.48	0.18	0.03

Rate constants k_1,_ k_2_ and k'_2_ are adjusted to satisfy rate Eq (10) which includes the F(t) correlation of the free water molecules.

Hydration water plays a significant role in all protein interactions with other molecules of biological interest. Therefore, it is very interesting to examine the behavior of HBs between protein and its surrounding water molecules (PW) in the presence of the field. A visual inspection of Fig 8B shows systematic differences between the number of protein-water HBs at different field conditions calculated for the last 10 ns of the 100 ns trajectories. Accordingly, Table 2 shows statistically significant decreases relative to zero field at f_2_, f_3_ (δ-relaxation) and f_4_ (γ-relaxation), with the largest (4%) observed at f_4_. For the same reason as in Table 1 the standard errors of the mean are once again very small (<0.24). Moreover, inspecting the protein-water HBs fluctuations (Fig 9B), it seems that at frequencies f_2_ and f_4_ the deviations from the mean converge at earlier times and to lower values compared to the behavior describing f_1_ and f_3_ and in the absence of the field.

Differences are more clear in WS1 inter-water hydrogen bonds. There is a small but distinguishable trend of the field to decrease the number of inter-water HBs (Table 2), with the largest decrease observed at f_4_ (9.6%). Since PW-HBs are formed between protein and water molecules belonging to S1, it might be expected that inter-water HBs fluctuations in S1 would be related to those of PW-HBs (Fig 9B and 9C). However, they should not be same because S1 contains also water molecules which may not be hydrogen bonded to protein. Noticeably, the number of HBs (PW+WS1) per water molecule in S1 remains equal to 1.07 at all field conditions.

Another result related to the above picture concerns the impact of the field on the density of the first hydration layer. The volume of S1 water layer required for these calculations have been determined by approximating lysozyme as an ellipsoid [50]. A thickness of 3 Å has been assumed for S1, as determined by combined SAXS and SANS experiments [51]. Our findings suggest a higher density (13.6%) of the first hydration layer than that of bulk water (assumed to possess a density of 996.516 kg/m^3^ at 27°C), very close to experimental results (10%) derived using SAS [51]. Interestingly, we found that the presence of the field imparts a decrease of the excess density, with the lowest one observed at 2×10^10^ Hz (Table 5). This correlates with the lower number of PW and WS1 HBs at this frequency (Table 2).

**Table 5:** Densities of water in S1 and excess densities percentage Δ(S1:Bulk) at different frequencies and zero field conditions.

	Density [kg/m^3^]	
f [Hz]	S1	Δ(S1:Bulk) [%]*
**Zero Field**	1142	13.6
**f_1_ = 10^7^**	1134	12.9
**f_2_ = 3×10^8^**	1105	10.3
**f_3_ = 3×10^9^**	1067	6.8
**f_4_ = 2×10^10^**	1043	4.6

*Δ(S1:Bulk) is calculated relative to the expected density for bulk water at 27^ο^C (996.516 kg/m^3^).

The above frequency dependent changes may be explained more likely in terms of an interplay between two hypothetical mechanisms, direct field induced HB breaking and HB formation affected by field facilitated diffusion. Given that the density of S1 is normally higher than those in S2 and bulk, a higher water mobility induced by the field (lower friction) can result to a diffusion of S1 water molecules into the S2 or the bulk region. This would reduce the number and density of S1 water molecules, in accordance to our observations (Fig 8D, Table 5). A smaller number of S1 water molecules would reduce the probability of HBs formation between themselves and between protein and water (Table 2). As a consequence (at high frequencies) some of the charged and polar amino acids, which have lost their water partners, will form ion bridges and therefore PP HBs are favored (Table 2). On the other hand, at lower frequencies, the field seems to affect PP HBs more directly by reducing their number and thereby increasing PW HBs (Fig 8A and 8B, Table 2). The hypothesized higher mobility induced by the field is probably connected to a momentary increase in temperature, which is corrected by the thermostat in the next steps. Of course this hypothesis has to be verified and expressed in mathematical terms, which will be the task of next studies."

### Kinetics of hydrogen bond breaking and formation

The last part of this section deals with the dependence of the dynamical properties of system's hydrogen bonds on frequency. In recent years several simulation studies focused on the dynamics and kinetics of hydrogen bonds based on calculations of various correlations functions [52]. Concerning the first solvation shell S1 and various active sites of proteins, a different relaxation behavior of PW hydrogen bonds has been found due to the structural heterogeneity of the different protein segments [52]. In addition, in water simulations with application of a static electric field, it has been found that the kinetics of HBs are affected for field strengths above 100 mV/Å in agreement with theoretical findings, suggesting that the average number of hydrogen bonds remains constant up to the aforementioned field limit [53]. In the following paragraphs we analyze HBs kinetics at frequencies f_1_, f_2_, f_3_ and f_4_ and at zero field conditions in two hydrogen bond systems, protein-water and inter-water in S1.

#### Protein-water hydrogen bonds

**Fig 11:**
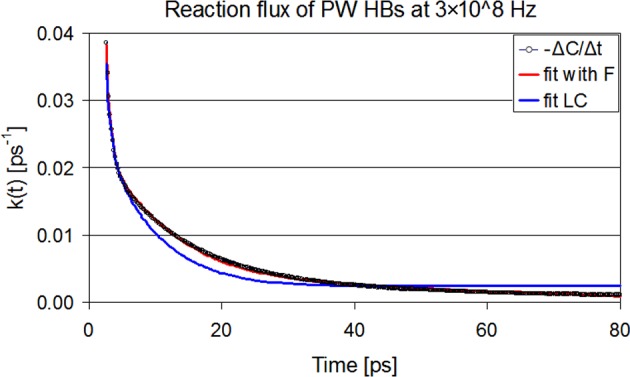
Protein-water hydrogen bond reactive flux k(t) at 3×10^8^ Hz. Points represent k(t) as -ΔC/Δt calculated from simulation data. Blue line represents the best fit that satisfies Eq (7), while the red one is a fit including the relaxation of F(t) correlation.

In order to make a comparison between the kinetics of bond breaking and reformation concerning the protein-water HBs network in the above conditions, we used Eqs 5b, 8 and 11 to calculate the time correlation C_PW_(t) for the intermittent HBs and the probability functions of broken hydrogen bonds N_PW_(t) and F_PW_(t), which are shown in Fig 10. The corresponding analytic expressions (four, three and two exponentials respectively) fit almost perfectly the simulation data and result to the weighted average relaxation times τ_C_^PW^, τ_N_^PW^ and τ_F_^PW^, which are listed in Table 3. As it is apparent from Fig 10 and Table 3, the field slows down the kinetic functions, with 3×10^8^ Hz and 3×10^9^ Hz showing the slowest relaxations in C(t) and N(t), while F(t) is slower than C(t) and N(t) at all frequencies, except f_2_. We recall that these frequencies correspond to the characteristic relaxations attributed to the hydration water and mobile protein segments. The clearest improvement in fit for reaction flux k(t) is observed at 3×10^8^ Hz after taking into account the F correlation, as shown in Fig 11. In fact, it is not easy to interpret the differences in the populations of HBs involved in C(t), N(t) and F(t) dynamics in relation to the applied field. The most tangible number regarding HBs kinetics is the HB life time calculated as ζ^PW^ = 1/k_1_ which is presented in Table 2. The largest acceleration effect of the field on HB breaking is observed at f_2_.

One may expect the kinetics of PW HBs to correlate with the induced by the field positional fluctuations along the protein. The frequency with the largest impact on the protein ΔRMSF is f_1_, while that with the lowest one is f_4_. However, HBs kinetics do not correlate directly with ΔRMSFs, because HBs breakings are a combined result of protein site fluctuations and water molecule rotations each with different frequency dependence.

#### S1 water hydrogen bonds

**Fig 12:**
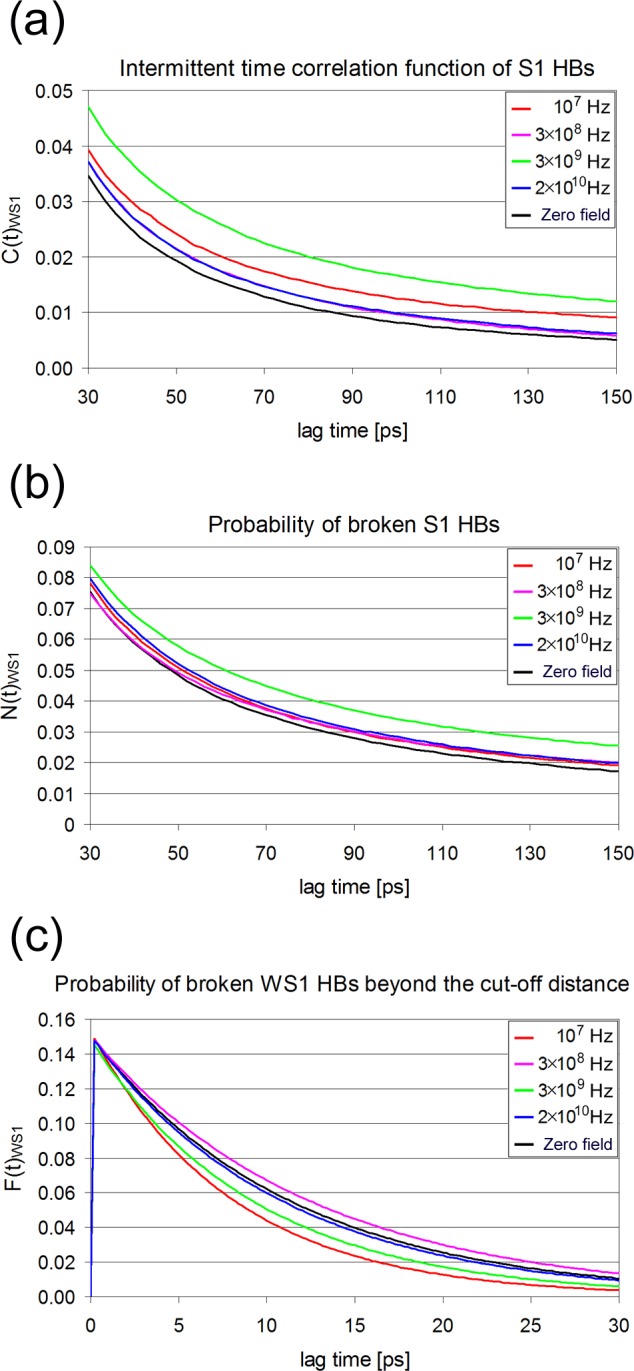
Correlation functions characterizing hydrogen bond breaking in the first hydration shell at different frequencies at 300 K. For better visualization of the differences at longer times data are plotted between 30 and 150 ps. (a) Intermittent hydrogen bond time correlation function. (b) Correlation probability functions of broken hydrogen bonds. (c) Correlation probability functions of broken inter-water hydrogen bonds between water molecules located slightly beyond the cut-off distance.

In this section we follow the previous analysis in order to study the kinetics of HBs between the water molecules of the first hydration shell (S1). As in the PW case, after an initial breaking of HBs between a target S1 water molecule with its neighbors, some of the neighboring molecules can be found at a distance slightly greater that 3.5 Å, still having the possibility to reform directly the initial HB after few simulation steps. The time correlation functions for the intermittent hydrogen bond C_WS1_(t) and the probability of broken hydrogen bonds N_WS1_(t) and F_WS1_(t) are depicted in Fig 12. The corresponding average life times are listed in Table 4. A first observation is that WS1 kinetics are faster than those of PW. It seems that breaking of hydrogen bonds between S1 water molecules is 5–10 times faster than between protein and water. The N(t) kinetic is about 1.5 times faster in the WS1 system, resulting to an inverse relationship between C(t) and N(t). Apparently, rotational jumps of water molecules responsible for HB breaking are more frequent in inter-water HBs than in PW, because the protein sites are less mobile. We recall that a subset of water molecules involved in PW HBs are involved also in inter-water HBs. As it is apparent from Fig 12, the values of the above correlation functions vary with frequency but not significantly. Nevertheless, the slowest C(t) and N(t) relaxations occur at 3×10^9^ Hz, whereas F(t) at 3×10^8^ Hz. Again, the incorporation of F(t) improves the fits. Concerning the HB life times ζ^WS1^, there is no noticeable effect of the field (Table 2).

## Conclusions

This work has demonstrated that the dielectric spectrum of lysozyme calculated using an oscillating electric field at different frequencies, is well described by the Fröhlich-Kirkwood approach in the framework of the linear response theory. This result could be used as a criterion for choosing the maximum acceptable field strengths for similar calculations. The observed frequency dependent non-thermal effects on structure as well as on protein hydrogen bonds, as were described in the present study, provide new insight which can be useful in the prediction of the protein’s function under similar conditions. Regarding the first hydration shell, our findings suggest a field-dependent water density as well as frequency-dependent changes in the rates of hydrogen bond breaking and reforming between protein and water. Concerning the later, the L&C model has been extended to describe better the hydrogen bond kinetics realized in field frequencies within the microwave range. The results of this work can be used as a starting point for a more detailed interpretation of future experimental studies in such systems, in the time or the frequency domain (e.g., neutron scattering).

## Supporting Information

S1 Table Table A. Values of the dielectric function. Table B. Weighted average relaxation times of PW HBs. Table C. Relaxation times of S1 HBs. Table D. Hydrogen bond breaking rate constants k_1_, k_2_ and k'_2_ for PW HBs. Table E. Hydrogen bond breaking rate constants k_1_, k_2_ and k'_2_ for S1 inter-water HBs.
(DOCX)

S1 Fig Figure A. Number of intra protein HBs at different frequencies. Figure B. Number of protein-water HBs at different frequencies. Figure C. Number of interwater HBs in S1 at different frequencies.
(TIF)

## References

[1] Miura N, Asaka N, Shinyashiki N, Mashimo S. Microwave dielectric study on bound water of globule proteins in aqueous solution. Biopolymers. 1994; 34: 357–364.

[2] Bonicontro A, Calandrini V, Onor G. Rotational and translational dynamics of lysozyme in water–glycerol solution. Colloids Surf B Biointerfaces. 2001; 21: 311–316. 11397633 10.1016/s0927-7765(00)00214-9

[3] Yokoyama K, Kamei T, Minami H, Susuki M. Hydration study of globular proteins by microwave dielectric spectroscopy. J Phys Chem. 2001; 105: 12622–12627.

[4] Knocks A, Weingartner H. The Dielectric Spectrum of Ubiquitin in Aqueous Solution J. Phys. Chem. 2001; 105: 3635–3638.

[5] Hayashi Y, Shinyashiki N, Yagihara S. Dynamical structure of water around biopolymers investigated by microwave dielectric measurements via time domain reflectometry. J Non-Crist Solids 2002; 305: 328–332.

[6] Cametti C, Marchetti S, Gambi C, Onon G. Dielectric relaxation spectroscopy of lysozyme aqueous solutions: analysis of the δ-dispersion and the contribution of the hydration water. J Phys Chem B. 2011; 115: 7144–7153. 10.1021/jp2019389 21557554

[7] Pethig R. Protein-water interactions determined by dielectric methods. Annu Rev Phys Chem. 1992; 43: 177–205. 10.1146/annurev.pc.43.100192.001141 1463572

[8] Nandi N and Bagchi B. Anomalous dielectric relaxation of aqueous protein solutions. J Phys Chem A. 1998; 102: 8217–8221.

[9] South G and Grant P. Dielectric dispersion and dipole moment of myoglobin in water. Proc. R. Soc. London Ser. A. Math. Phys. Sci. 1972; 328: 371–387.

[10] Suzuki M, Shigematsou J, Kodama T. Hydration study of proteins in solution by microwave dielectric analysis. J Phys Chem. 1996; 100: 7279–7282.

[11] Rosen A, Greenspon AJ, Walinsky P. Microwaves Treat Heart Disease. IEEE Microw Mag. 2007; 8(1): 70–75.

[12] Semenov S. Microwave tomography: review of the progress towards clinical applications. Philos Trans R Soc Lond A. 2009; 367: 3021–3042. 10.1098/rsta.2009.0092 PMC2696111 19581253

[13] Brace LC. Microwave Tissue Ablation: Biophysics, Technology, and Applications. Crit Rev Biomed Eng. 2010; 8(1): 65–78. 10.1615/critrevbiomedeng.v38.i1.60 PMC3058696 21175404

[14] Floros S, Liakopoulou-Kyriakides M, Karatasos K, Papadopoulos GE. Detailed study of the dielectric function of a lysozyme solution studied with molecular dynamics simulations. Eur Biophys J. 2015 12;44(8):599–611. 10.1007/s00249-015-1052-7 26094070

[15] Nakamura H, Sakamoto T, Wada A. A theoretical study of the dielectric constant of a protein. Protein Eng. 1988; 2: 177–183. 3237682 10.1093/protein/2.3.177

[16] King G, Lee FS, Warshel A. Microscopic simulations of macroscopic dielectric constants of solvated proteins. J Chem Phys. 1991; 95: 4366–4377.

[17] Simonson T.; Perahia D.; Brunger AT. Microscopic theory of the dielectric properties of proteins. Biophys J. 1991; 59(3): 670–690. 10.1016/S0006-3495(91)82282-2 1646659 PMC1281231

[18] Smith PE, Brunne RM, Mark AE, Van Gunsteren WF. Dielectric Properties of Trypsin Inhibitor and Lysozyme Calculated from Molecular Dynamics Simulations. J Phys Chem. 1993; 97: 2009–2014.

[19] Antosiewicz J, Mccammon J, Gilson M. Prediction of pH-dependent properties of proteins. J Mol Biol. 1994; 238: 415–436. 10.1006/jmbi.1994.1301 8176733

[20] Simonson T and Perahia D. Internal and interfacial dielectric properties of cytochrome c from molecular dynamics in aqueous solution. Proc Natl Acad Sci USA. 1995; 92(4): 1082–1086. 7862638 10.1073/pnas.92.4.1082 PMC42641

[21] Yang L, Weerasinghe S, Smith P, Pettitt B, Dielectric response of triplex DNA in ionic solution from simulations. Biophys J. 1995; 69: 1519–1527. 10.1016/S0006-3495(95)80022-6 8534822 PMC1236382

[22] Loffler G, Schreiber H, Steinhauser O. Calculation of the dielectric properties of a protein and its solvent: Theory and a case study. J Mol Biol. 1997; 270: 520–534. 10.1006/jmbi.1997.1130 9237916

[23] Boresch S, Ringhofer S, Hochtl P, Steinhauser O. Towards a better description and understanding of biomolecular solvation. Biophys Chem. 1999; 78: 43–68. 17030304 10.1016/s0301-4622(98)00235-x

[24] Boresch S, Hochtl P, Steinhauser O. Studying the dielectric properties of a protein solution by computer simulation. J Phys Chem B. 2000; 104(36): 8743–8752.

[25] Fröhlich H. In: Theory of dielectrics. Oxford Clarendon Press; 1958 Chapter I, pp. 8–9, Eq 2.20

[26] Kirkwood JG. The dielectric polarization of polar liquids. J Chem Phys. 1939; 7: 911–919

[27] Onsager L. Electric Moments of Molecules in Liquids. J Am Chem Soc. 1936; 58(8): 1486–1493.

[28] Neumann M, Steinhauser O, Pawley GS. Consistent calculation of the static and frequency-dependent dielectric-constant in computer-simulations. Mol Phys. 1984; 52(1): 97–113.

[29] Neumann M. Dielectric-relaxation in water—computer-simulations with the tip4p potential. J Chem Phys. 1986; 85: 1567–1580.

[30] Neumann M. Computer-simulation and the dielectric-constant at finite wavelength. Mol Phys. 1986; 57(1): 97–121.

[31] Kohler F. The liquid state. Weinheim Verlag Chemie; 1972.

[32] Neumann M, Steinhauser O. On the calculation of the frequency-dependent dielectric constant in computer simulations. Chem Phys Lett. 1983; 102: 508–513.

[33] Neumann M, Steinhauser O. On the calculation of the dielectric constant using the Ewald-Kornfeld tensor. Chem Phys Lett. 1983; 95: 417–422.

[34] Caillol JM, Levesque D, Weis JJ. Theoretical calculation of ionic solution properties. J Chem Phys. 1986; 85: 6645–6657.

[35] Caillol JM, Levesque D, Weis JJ. Electrical properties of polarizable ionic solutions. I. Theoretical aspects J Chem Phys. 1989; 91: 5544–5554.

[36] Weingartner H, Knocks A, Boresch S, Hochtl P, Steinhauser O. Dielectric spectroscopy in aqueous solutions of oligosaccharides: Experiment meets simulation. J Chem Phys. 2001; 115(3): 1463–1472.

[37] English JN and Mooney AD. Denaturation of hen egg white lysozyme in electromagnetic fields: A molecular dynamics study. J Chem Phys. 2007; 126: 091105 10.1063/1.2515315 17362097

[38] Niall J, Einglish GY, Sollomentsev PO. Nonequilibrium molecular dynamics study of electric and low-frequency microwave fields on hen egg white lysozyme. J Chem Phys. 2009; 131: 035106 10.1063/1.3184794 19624238

[39] Rapaport DC. Hydrogen bonds in water Network organization and lifetimes. Mol Phys. 1983; 50: 1151–1162.

[40] Luzar A and Chandler D. Effect of environment on hydrogen bond dynamics in liquid water. Phys Rev Lett. 1996; 76: 928–931. 10.1103/PhysRevLett.76.928 10061587

[41] Luzar A. Resolving the hydrogen bond dynamics conundrum. J Chem Phys. 2000; 113: 10663–10675.

[42] Luzar A. Extent of inter-hydrogen bond correlations in water. Temperature effect. Chem Phys. 2000; 258: 267–276.

[43] Kale L, Skeel R, Bhandarkar M, Brunner R, Gursoy A, Krawetz N, et al NAMD2: Greater scalability for parallel molecular dynamics. J Comput Phys. 1999;151(1): 283–312.

[44] Brooks R, Bruccoleri RE, Olafson BD, States DJ, Swaminathan S, Karplus M. CHARMM: A program for macromolecular energy, minimization, and dynamics calculations. J Comput Chem. 1983; 4(2): 187–217.

[45] Ryckaert JP, Ciccotti G, Berendsen HJC. Numerical integration of the cartesian equations of motion of a system with constraints: molecular dynamics of n-alkanes. J Comput Phys. 1977; 23(3): 327–341.

[46] Darden T, York D, Pedersen L. Particle mesh Ewald: An N·log(N) method for Ewald sums in large systems. J Chem Phys. 1993; 98: 10089–10092.

[47] NAMD User's Guide Version 2.9. 2012; Available: http://www.ks.uiuc.edu/Research/namd/2.9/ug/.

[48] Humphrey W, Dalke A, Schulten K. VMD—Visual Molecular Dynamics. J Mol Graph. 1996; 14(1): 33–38. 8744570 10.1016/0263-7855(96)00018-5

[49] Barteri M, Pala A, Rotella S. Structural and kinetic effects of mobile phone microwaves on acetylcholinesterase activity. Biophys Chem. 2005; 113: 245–253. 10.1016/j.bpc.2004.09.010 15620509

[50] Muller JJ and Schrauber H. The Inertia-Equivalent Ellipsoid: a Link Between Atomic Structure and Low-Resolution Models of Small Globular Proteins Determined by Small-Angle X-Ray Scattering. J Appl Cryst. 1992; 25: 181–191.

[51] Svergun DI, Richard S, Koch MHJ, Sayers Z, Kuprin S and Zaccai G. Protein hydration in solution: Experimental observation by x-ray and neutron scattering. Proc Natl Acad Sci USA Biophysics. 1998; 95: 2267–2272. 10.1073/pnas.95.5.2267 PMC19315 9482874

[52] Bandyopadhyay S, Chakraborty S, and Bagchi B. Secondary Structure Sensitivity of Hydrogen Bond Lifetime Dynamics in the Protein Hydration Layer. J Am Chem Soc. 2005; 127: 16660–16667. 10.1021/ja054462u 16305255

[53] Suresh SJ and Satish AV. Influence of electric field on the hydrogen bond network of water. J Chem Phys. 2006; 124: 074506. 10.1063/1.2162888 16497056

