# Supplementary material for: Frequency Dependent Non- Thermal Effects of Oscillating Electric Fields in the Microwave Region on the Properties of a Solvated Lysozyme System: A Molecular Dynamics Study
Source: PLoS One. 2017 Jan 27;12(1):e0169505. doi: 10.1371/journal.pone.0169505 (PMC5271316; doi:10.1371/journal.pone.0169505)
Supplement: S1 Table — Table A. Values of the dielectric function. Table B. Weighted average relaxation times of PW HBs. Table C. Relaxation times of S1 HBs. Table D. Hydrogen bond breaking rate constants k1, k2 and k'2 for PW HBs. Table E. Hydrogen bond breaking rate constants k1, k2 and k'2 for S1 inter-water HBs. (DOCX) [file pone.0169505.s001.docx]

**Table A. Dielectric function values.** Values of the dielectric function for protein and solution at 21 different frequencies derived from the Fröhlich-Kirkwood approach (FK) and from direct application of electric fields (EF). For the EF values standard errors of the fit procedure are given. The last column shows the number of independent simulations used.

| f [Hz] | ε^’^_p_ FK  ε^’^_p_ EF | ε^’’^_p_ FK  ε^’’^_p_ EF | ε^’^ FK  ε^’^ EF | ε^’’^ FK  ε^’’^ EF | N_SIM |
| --- | --- | --- | --- | --- | --- |
| 10^7^ | 9.36  9.30 ±0.094 | 7.78  7.76 ±0.094 | 78.96  78.10 ±0.274 | 7.87  8.14 ±0.274 | 1 |
| 5×10^7^ | 2.88  3.00 ±0.010 | 2.87  2.73 ±0.010 | 72.81  71.04 ±0.064 | 3.62  4.18 ±0.064 | 2 |
| 10^8^ | 2.45  2.39 ±0.016 | 1.52  1.45 ±0.016 | 72.09  71.87 ±0.078 | 3.18  3.05 ±0.078 | 3 |
| 10^0.5^×10^8^ | 2.29  2.34 ±0.004 | 0.62  0.64 ±0.004 | 70.63  69.85 ±0.027 | 3.90  3.76 ±0.027 | 8 |
| 10^9^ | `1.97  2.12 ±0.003 | 0.54  0.48 ±0.003 | 68.12  67.62 ±0.025 | 5.82  5.64 ±0.025 | 14 |
| 10^0.2^×10^9^ | 1.94  2.01 ±0.002 | 0.58  0.52 ±0.002 | 67.09  66.93 ±0.022 | 7.035  6.74 ±0.022 | 16 |
| 10^0.4^×10^9^ | 1.71  1.70 ±0.002 | 0.58  0.56 ±0.002 | 66.10  66.66 ±0.024 | 9.16  8.94 ±0.024 | 18 |
| 10^0.6^×10^9^ | 1.50  1.54 ±0.003 | 0.50  0.52 ±0.003 | 64.57  65.21 ±0.029 | 12.77  12.51 ±0.029 | 18 |
| 10^0.8^×10^9^ | 1.35  1.33 ±0.005 | 0.38  0.40 ±0.005 | 61.48  60.88 ±0.042 | 18.06  18.05 ±0.042 | 18 |
| 10^0.9^×10^9^ | 1.30  1.32 ±0.002 | 0.31  0.34 ±0.002 | 58.75  57.44 ±0.039 | 21.73  21.67 ±0.039 | 19 |
| 10^10^ | 1.27  1.36 ±0.002 | 0.26  0.28 ±0.002 | 55.26  54.70 ±0.066 | 24.56  24.54 ±0.066 | 19 |
| 10^0.1^×10^10^ | 1.24  1.30 ±0.001 | 0.21  0.22 ±0.001 | 50.50  51.23 ±0.029 | 27.65  27.30 ±0.029 | 20 |
| 10^0.2^×10^10^ | 1.23  1.33 ±0.001 | 0.17  0.19 ±0.001 | 44.74  43.77 ±0.017 | 29.99  29.22 ±0.017 | 20 |
| 10^0.3^×10^10^ | 1.22  1.30 ±0.002 | 0.13  0.17 ±0.002 | 38.10  38.75 ±0.023 | 31.19  31.08 ±0.023 | 20 |
| 10^0.4^×10^10^ | 1.21  1.25 ±0.003 | 0.11  0.15 ±0.003 | 31.31  30.81 ±0.037 | 30.92  30.31 ±0.037 | 20 |
| 10^0.5^×10^10^ | 1.21  1.20 ±0.003 | 0.08  0.11 ±0.003 | 24.80  23.96 ±0.048 | 29.20  29.14 ±0.048 | 20 |
| 10^0.6^×10^10^ | 1.21  1.17 ±0.006 | 0.07  0.10 ±0.006 | 19.26  18.23 ±0.088 | 26.44  26.21 ±0.088 | 20 |
| 10^0.7^×10^10^ | 1.21  1.13 ±0.002 | 0.05  0.08 ±0.002 | 14.89  13.45 ±0.027 | 23.05  22.56 ±0.027 | 25 |
| 10^0.8^×10^10^ | 1.21  1.09 ±0.003 | 0.04  0.07 ±0.003 | 11.64  10.77 ±0.034 | 19.56  18.98 ±0.034 | 25 |
| 10^0.9^×10^10^ | 1.20  1.00 ±0.003 | 0.03  0.06 ±0.003 | 9.33  8.24 ±0.038 | 16.27  15.85 ±0.038 | 25 |
| 10^11^ | 1.20  1.00 ±0.003 | 0.02  0.05 ±0.003 | 7.76  6.81 ±0.046 | 13.40  13.15 ±0.046 | 26 |

**Table B. Weighted average relaxation times of PW HBs.** Weighted average relaxation times τ^PW^ of PW HBs correlation functions C_PW_(t), N_PW_(t) and F_PW_(t) at four characteristic frequencies and at zero field.

| f [Hz] | τ_C_^PW^ [ps] | E_rms_ | τ_N_^PW^ [ps] | E_rms_ | τ_F_^PW^ [ps] | E_rms_ |
| --- | --- | --- | --- | --- | --- | --- |
| Zero field | 34.4 | 0.00036 | 14.9 | 0.00015 | 43.7 | 0.00171 |
| f1 = 10^7^ | 42.2 | 0.00039 | 16.7 | 0.00015 | 47.6 | 0.00120 |
| f2 = 3×10^8^ | 56.4 | 0.00039 | 20.5 | 0.00016 | 48.1 | 0.00124 |
| f3 = 3×10^9^ | 52.2 | 0.00031 | 22.4 | 0.00017 | 60.4 | 0.00111 |
| f4 = 2×10^10^ | 47.6 | 0.00031 | 17.9 | 0.00020 | 52.0 | 0.00109 |

**Table C.** **Relaxation times of S1 HBs.** Relaxation times τ of S1 HBs correlation functions C_WS1_(t), N_WS1_(t) and F_WS1_(t) at four characteristic frequencies and at zero field.

| f [Hz] | τ_C_^WS1^ [ps] | E_rms_ | τ_N_^WS1^ [ps] | E_rms_ | τ_F_^WS1^ [ps] | E_rms_ |
| --- | --- | --- | --- | --- | --- | --- |
| Zero field | 5.1 | 0.00021 | 10.6 | 0.00032 | 1.7 | 0.00396 |
| f1 = 10^7^ | 6.9 | 0.00027 | 11.4 | 0.00037 | 1.2 | 0.00305 |
| f2 = 3×10^8^ | 5.3 | 0.00020 | 11.6 | 0.00034 | 1.8 | 0.00359 |
| f3 = 3×10^9^ | 8.1 | 0.00025 | 13.8 | 0.00040 | 1.4 | 0.00313 |
| f4 = 2×10^10^ | 5.8 | 0.00027 | 11.7 | 0.00031 | 1.6 | 0.00328 |

Estimates of the fit quality are given using E_rms_.

$$E_{rms}=\sqrt{\frac{\sum_{1}^{N} \left[X_{n}-Y_{n}\right]^{2}}{N-df}},$$

The time constants τ_C_, τ_N_ and τ_F_ in tables B and C are calculated from the corresponding fitted C, N and F correlation functions. E_rms_ are calculated according to the above equation for the C, N and F fits. X_n_ are values from the simulations, while Y_n_ those from the fitted analytic functions at lag time step n. N is the total number of lag time steps and df is the corresponding degrees of freedom.

**Table D.** **Hydrogen bond breaking rate constants k_1_, k_2_ and k'_2_ for PW HBs.**

| f [Hz] | k_1_^PW^ [ps^-1^] | k_2_^PW^ [ps^-1^] | k’_2_^PW^ [ps^-1^] | E_rms_ |
| --- | --- | --- | --- | --- |
| Zero field | 0.338 ± 0.0005 | 0.697 ±0.0015 | 0.036 ± 9×10^-5^ | 0.000132 |
| f1 = 10^7^ | 0.408 ± 0.0025 | 0.878 ±0.0066 | 0.049 ±0.0006 | 0.000382 |
| f2 = 3×10^8^ | 1.138 ±0.0090 | 2.777 ± 0.0248 | 0.067 ±0.0007 | 0.000121 |
| f3 = 3×10^9^ | 0.410 ±0.0033 | 0.740 ±0.0086 | 0.120 ±0.0013 | 0.000769 |
| f4 = 2×10^10^ | 0.929 ±0.0032 | 2.293 ±0.0913 | 0.031 ±0.0004 | 0.000114 |

**Table E.** **Hydrogen bond breaking rate constants k_1_, k_2_ and k'_2_ for S1 inter-water HBs.**

| f [Hz] | k_1_^WS1^ [ps^-1^] | k_2_^WS1^ [ps^-1^] | k’_2_^WS1^ [ps^-1^] | E_rms_ |
| --- | --- | --- | --- | --- |
| Zero field | 0.51 ±0.012 | 0.18 ±0.005 | 0.04 ±0.002 | 0.000884 |
| f1 = 10^7^ | 0.69 ±0.016 | 0.31 ±0.007 | 0.05 ±0.001 | 0.000846 |
| f2 = 3×10^8^ | 0.49 ±0.010 | 0.19 ±0.005 | 0.03 ±0.001 | 0.000793 |
| f3 = 3×10^9^ | 0.61 ±0.015 | 0.30 ±0.008 | 0.03 ±0.002 | 0.000852 |
| f4 = 2×10^10^ | 0.48 ±0.011 | 0.18 ±0.005 | 0.03 ±0.002 | 0.000817 |

For the k values in tables D and E standard errors of the fit procedures are given. The last column shows the E_rms_, with X_n_ = (-dC/dt)_n_ from simulation data, while Y_n_ = k_1_C_n_ - k_2_N_n_ -k_3_F_n_.
