# Supplementary figures and images for: Frequency Dependent Non- Thermal Effects of Oscillating Electric Fields in the Microwave Region on the Properties of a Solvated Lysozyme System: A Molecular Dynamics Study
Source: PLoS One. 2017 Jan 27;12(1):e0169505. doi: 10.1371/journal.pone.0169505 (PMC5271316; doi:10.1371/journal.pone.0169505)

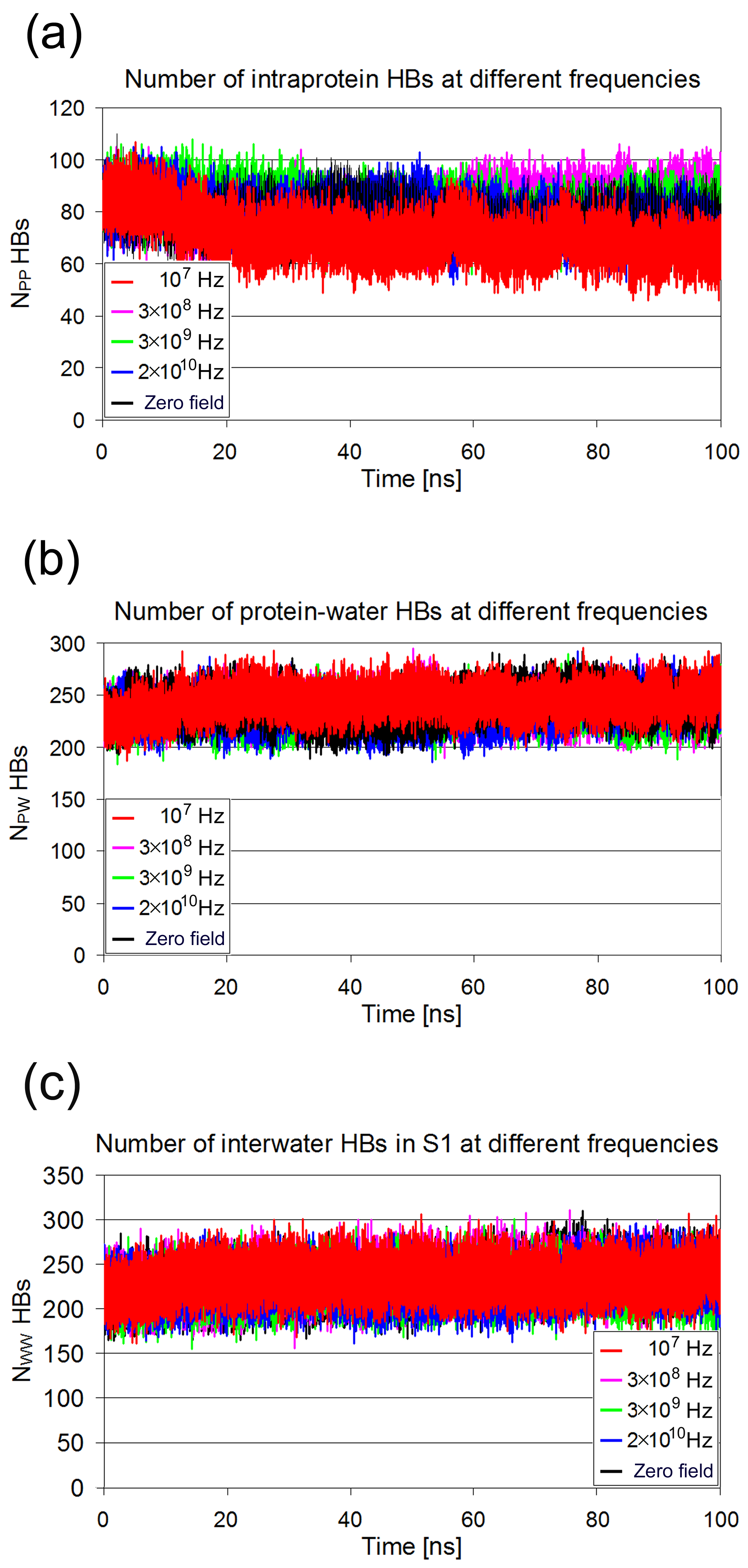

Supplement: S1 Fig — Figure A. Number of intra protein HBs at different frequencies. Figure B. Number of protein-water HBs at different frequencies. Figure C. Number of interwater HBs in S1 at different frequencies. (TIF) [file pone.0169505.s002.tif]
